# To Prevent the Spreading of Scarlet Fever

**Published:** 1881-04

**Authors:** J. Davidson


### TO PREVENT THE SPREADING OF SCARLET FEVER.

J. DAVIDSON, M. D.

The following rules have received the sanction of the highest medical authorities, and when faithfully carried out have been found effectual in limiting the contagiousness of scarlet fever. At the same time it should be remembered that it is also epidemic as well as contagious, and cases frequently occur where contact has not taken place:

1st. Prompt removal of the healthy from sick children, even members of the same family, as far as possible.

2d. The destruction or complete disinfection of every article that has come in contact with the sick.

3d. If possible, the patient should have a separate room, into which none but nurses are admitted.

4th. Remove from the room useless furniture, as musical instruments, book cases, cupboards, and everything which can collect dust and dirt, as window curtains, and above all woolen or heavy drapery, carpets, etc. The contagious germs are deposited as a fine dust, on woolen goods and carpets especially, and will retain the vitality of the poisonous germs for a long period. Never allow the patient to expectorate on the floor or on carpets; use spittoons containing adisinfecting fluid. The secretions of the mouth and nostrils are heavily charged with poisonous germs, and when dry and deposited on the floor or carpets, have the power of reinfecting almost indefinitely.

5th. Ventilate the room by an open window at the top, or if very cold weather, ventilate the adjoining room, the door between being left open, but protect the patient from direct drafts of air.

6th. Keep the patient clean, changing under clothing often, and every article used by him should be thoroughly disinfected. The expectoration and other discharges should be received into vessels containing disinfecting fluids. Chloride of Lime, one-half pound to a pail of water; or carbolic acid, two ounces to pail of water, and should be immediately removed and placed buried. The underclothing should be in one of the above fluids for an hour or two, and then washed in very hot water in tubs used only for this purpose. Water at the boiling point promptly kills the fever germs.

7th. Instead of using pocket handkerchiefs about the patient use pieces of cotton or linen, and burn them when soiled.

8th. The sweepings and dustings of the room should be destroyed by fire.

9th. The nurse's clothes and hands should be disinfected and washed in one of the fluids as above, frequently.

10th. Dry the clothing after washing with a high degree of heat, and then give them a thorough airing in the cold air. Extremes of heat and cold destroy the fever germs.

11th. The convalescent should not mingle with the healthy in less time than a month from the beginning of the attack. The room he has occupied should be thoroughly cleaned and disinfected, and re-papered or painted, and the windows and doors be allowed to remain open a long time.

12th. The patients should be separated as much as possible from each other in the same house, as they re-infect each other and add to its malignancy. Deny to all children admittance to the house, and all visitors except nurses, until the complete disappearance of all symptoms of disease of the throat and skin.

Finally, all display should be prohibited at the funerals of those who have died of scarlet fever. Children should not be allowed to be there, and the opening of the coffin in the presence of friends should be avoided.

In conclusion, if these rules are observed at the homes of the sick, healthy adults, with no family of small children at home, need have no fear in giving aid and nursing in afflicted families, as scarlet fever in the adult is a very mild disease, especially if the subject has had it in childhood. But avoid coming in contact with young children, nevertheless. If you cannot assist the afflicted at their homes, you may furnish means to the poor in assisting them to carry out the above means of prevention.

